# Hair Testosterone and Cortisol Concentrations in Pre- and Post-Rut Roe Deer Bucks: Correlations with Blood Levels and Testicular Morphometric Parameters

**DOI:** 10.3390/ani8070113

**Published:** 2018-07-06

**Authors:** Domenico Ventrella, Alberto Elmi, Francesca Barone, Giacomo Carnevali, Nadia Govoni, Maria Laura Bacci

**Affiliations:** Department of Veterinary Medical Sciences, University of Bologna, 40064 Ozzano dell’Emilia (BO), Italy; domenico.ventrella2@unibo.it (D.V.); francesca.barone7@unibo.it (F.B.); giacomo.carnevali2@unibo.it (G.C.); nadia.govoni@unibo.it (N.G.); marialaura.bacci@unibo.it (M.L.B.)

**Keywords:** roe deer, hair testosterone, hair cortisol, testicular morphometric parameters, reproductive physiology, radioimmunoassay

## Abstract

**Simple Summary:**

The roe deer is a very common wild species in Italy and shows peculiar reproductive characteristics. Sexually-mature males, called bucks, show a complete interruption in spermatogenesis during the cold seasons. The mechanisms behind such interruption are still partially unknown. Hair is a good biological sample, easy to obtain while minimizing stress, for endocrinological analyses that may provide information regarding such mechanisms. The aim of the work was to quantify and compare hair concentrations of testosterone and cortisol in wild roe deer bucks hunted during the pre- and post-rut period (mating season). The secondary objective was the evaluation of possible correlations of such hair concentrations with blood and morphometric parameters of the testes. Testosterone significantly increased from the pre- to post-rut period, while cortisol significantly decreased. The correlations with blood and testicular parameters resemble what is already described in the literature. Overall, this study represents a first report of the quantification of testosterone and cortisol in roe deer hair, and may provide interesting insights into their reproductive physiology.

**Abstract:**

The roe deer is a seasonally breeding species with a reproductive cycle regulated by endogenous rhythms and photoperiod-sensitivity. Sexually mature bucks show hormonal and testicular activation during the reproductive season, with a peak in the rut period, and following gradual involution. Hair is a good matrix for non-invasive endocrinological analyses that provide long-term information without being influenced by the hormones’ pulsating release patterns in blood. The aim of the work was to quantify hair concentrations of testosterone and cortisol in wild roe deer bucks hunted during the pre- and post-rut period, using a radioimmunoassay methodology, and to look for differences between the two periods. The secondary objective was the evaluation of possible correlations of such hair concentrations with blood and morphometric parameters of the testes. Both hormones showed statistical differences, with opposing trends, when comparing the two periods: testosterone increased while cortisol decreased. The correlation analysis was in agreement with existing literature regarding metabolism/actions of these hormones and testicular morphometric parameters. This study represents the first report of the use of radioimmunoassay techniques to quantify testosterone and cortisol in roe deer hair, and may provide interesting insights into their reproductive physiology.

## 1. Introduction

Mammalian species living in the Northern hemisphere have developed different strategies in order to ensure successful breeding as an adaptation to the cycling environmental conditions of their habitats [1]. A good example of this adaptive process can be found in the seasonal reproductive cycle of the roe deer (*Capreolus capreolus* L., 1758), a European wild ruminant. Its reproductive season is characterized by three periods: pre-rut (mid-May/mid-July), rut (mid-July/mid-August) and post-rut (mid-August/September) [2], and is regulated by endogenous rhythms and high sensitivity to photoperiod [1]. Right before the pre-rut period (from the beginning of April), bucks show aggressive behaviors as they fight each other in order to establish hierarchy and territoriality [3]. In the same period, the first of the two annual sheddings takes place, with the complete substitution of the winter coat [4]. Once sexually mature, bucks show highly synchronized testicular cycles including transitions from totally arrested to highly activated spermatogenesis [5]. Spermatogenesis is directly linked to growth and involution patterns of the testicular mass [6]; indeed, during the rut period, an increase in diameters of the seminiferous tubules (up to 220 µm), and a proliferation of spermatogenic cells is observed [1]. On the other hand, at the end of the reproductive season, spermatogenesis is inactivated with seminiferous tubules that decrease in diameter up to 90 µm and lower cell density; at this stage, spermatogonia and Sertoli cells represent the only epithelial cellular components of the testis [7]. These cycling reproductive patterns are strictly related to blood and intra-testicular levels of steroid hormones, in particular testosterone (TEST): high levels are indeed necessary for successful spermatogenesis, good functionality of accessory sex glands, and mating outcome [1]. In bucks, TEST reaches its intra-testicular [6] and peripheral blood [8] peaks during the rut period, and decreases to basal levels starting from October [5]. The same trend applies to other sexual steroids including progesterone, androstenedione, and estradiol [8]. Another important steroid hormone is cortisol (CORT), generally used as stress marker for wildlife animals alongside corticosterone [9], since stress can impair reproductive efficiency and performances. One of the biggest limitations to hematic CORT quantification lies in the ultra-fast activation of the hypothalamic-pituitary-adrenocortical (HPA) system during stressful events [10], making it almost useless when assessing environmental chronic stressors. This is the reason why in the last decades, alternative biological matrices for CORT and other stress-related hormones quantification were investigated, including hair and feces [11,12]. Amongst the others, hair proved to be one of the best, if not the best, matrix to gain information regarding chronic and environmental stressors in different mammalian species [13,14,15,16,17], including humans [18]. Indeed, it is relatively easy and non-invasive to collect and less subject to deterioration [19]. Regarding the roe deer, alternative matrices such as feces were used to assess CORT concentrations [20], but hair, to the best of the authors’ knowledge, has never been taken into account. In the light of the cycling reproductive pattern of this species and its strong relationship with the environment, the evaluation of hair steroid hormones may provide interesting insights to further characterize this process. Therefore, the aim of the present work was to quantify hair concentrations of testosterone and cortisol in wild roe deer bucks hunted during either pre- or post-rut period, and to compare the results. The secondary objective was the evaluation of any possible correlations of such hair concentrations with blood and morphometric parameters of the testes.

## 2. Materials and Methods

### 2.1. Animals and Sampling

Twenty-eight sexually mature roe deer bucks, 1 to 7 years old with body weights between 20.0 and 31.4 kg, were sampled during the 2017 hunting season between 1 June and 15 July (pre-rut, *n* = 14) and 15 August and 30 September (post-rut, *n* = 14). Animals were hunted in the South-Western Bologna Apennines (Italy) according to the regional hunting plan (Resolution No. 473 of the Emilia Romagna Regional Executive, 10 April 2017). Upon death, all the animals were immediately transferred to the pertinent biometrical center. Animals found dead within the territory during the hunting periods were not included in the study to avoid biases related to possible pathological conditions. Ages were assessed upon analyses of teeth eruption and wear patterns.

Upon arrival, the personnel of the centers collected blood from the jugular vein in sterile Lithium-heparin tubes, hair from the dorsal-caudal region and scrotums, including testes and epididymis. The samples were refrigerated (5 ± 1 °C) and transferred, using a cooler to maintain temperature, within 24 h to the physiology laboratories (ANFI-ASA) of the Department of Veterinary Medical Sciences of the University of Bologna (Ozzano dell’Emilia, Italy) [6]. Blood was centrifuged for 15 min at 1500× *g* to separate plasma which was divided into aliquots and stored at −20 ± 2 °C. It was previously demonstrated how steroids are stable in whole blood if kept refrigerated until centrifugation, which in the present case happened within 24 h [21,22]. Hair samples were stored at 5 ± 1 °C until analyses, while scrotums were immediately evaluated. Blood samples showing macroscopical alterations such as clots and scrotums damaged during collection were excluded from the study.

Since all the biological specimens analyzed in this study were obtained from hunted animals (in accordance with the hunting plan in force), no ethical approval was necessary.

### 2.2. Testicular Analysis

Testes were isolated from the scrotums and weighed using a lab scale (FCB 12K1, KERN & SOHN GmbH, Balingen, Germany) after epididymis ablation. For each animal, the weights of the two testes were averaged. 

To perform the morphometric evaluations, each testis was sectioned longitudinally and the major and minor axes were measured. The axes measures of the two testes were averaged for each roe deer. Finally, the mean value between the major and minor axes of the single animal was divided by two and used as the radius (*r*). To get the final measurement of the testicular volume (*V*), the following formula was applied:

$$V=4\pi \;\left(r^{3}/3\right)$$

### 2.3. Steroids Extraction from Plasma and Hair

For plasma, steroids were extracted by mixing 0.2 mL of each sample with 5 mL of diethyl ether (Merck KGaA, Darmstadt, Germany) for 30 min on a rotary mixer as previously described [23]. Tubes were then centrifuged at 2000× *g* for 15 min and the supernatants evaporated to dryness at 37 °C in a fume hood.

Hair samples were handled and analyzed as previously described by Bacci et al. [14]. Briefly, hair was washed with water and propanol-2-ol (Carlo Erba Reagents s.r.l., Cornaredo, Italy) in order to remove any organic residue from the surface. Once fully dried, hair was finely pulverized, and 180 mg of each pulverized sample was incubated overnight with 6 mL of methanol (Carlo Erba Reagents s.r.l., Cornaredo, Italy) for steroids extraction. After centrifugation at 1500× *g* for 30 min, methanol was collected and evaporated to dryness under an air-stream suction hood.

### 2.4. Radioimmunoassay (RIA) for Testosterone and Cortisol

The dry extracts were stored at −20 ± 2 °C until reconstitution in assay buffer for measurement of TEST (20 µL plasma equivalent, 28 mg hair equivalent) or CORT (10 µL plasma equivalent, 28 mg hair equivalent) by radioimmunoassay. Tritiated TEST (30 pg/100 µL; 83.4 Ci/mmol; PerkinElmer Inc., Boston, MA, USA) or tritiated CORT (30 pg/100 µL; 94.6 Ci/mmol PerkinElmer Inc., Boston, MA, USA) were added, followed by rabbit anti-TEST serum (0.1 mL, 1:50,000; antiserum produced in our laboratory) or rabbit anti-CORT serum (0.1 mL, 1:20,000; produced in our laboratory), respectively. After incubation and separation of antibody-bound and antibody-unbound steroid by charcoal-dextran solution (charcoal 0.25%, dextran 0.02% in phosphate buffer), tubes were centrifuged (15 min, 3000× *g*), the supernatant was decanted and radioactivity immediately measured using a β-scintillation counter (Packard C1600, Perkin Elmer, Boston, MA, USA).

The sensitivity of the TEST assay was 1.70 pg/100 µL, and the intra-assay coefficients of variation were 5.7%. The sensitivity of the CORT assay was 5.18 pg/100 µL, and the intra-assay coefficients of variation were 4.9%. Cross-reactions of various steroids with antiserum raised against TEST were: testosterone (100%), dihydrotestosterone (25.44%), androstenedione (0.6%), progesterone, and cortisol (<0.0001%). Cross reactions of various steroids with antiserum raised against CORT were: cortisol 100%, cortisone 5.3%, 11α-deoxycortisol 5.0%, corticosterone 9.5%, 20α-dihydrocortisone 0.4%, prednisolone 4.60%, progesterone <0.001%, and testosterone <0.001%.

In order to determine the parallelism between hormone standards and endogenous hormones in roe deer hair, a pooled sample containing high concentrations in CORT and TEST was serially diluted (1:1–1:8) with assay buffer. A regression analysis was used to determine parallelism between the two hormone levels in the same assay. A high degree of parallelism was confirmed by regression test (r^2^ = 0.98, *p* < 0.01). The recovery of extraction was performed on five replicates by adding 250, 500, or 1000 pg of 3H-testosterone or 3H-cortisol to 180 mg of hair, incubating for 18 h and extracting as described above. The mean percentage of recovery was 94.6 ± 2.4% and 94.3 ± 1.8%, respectively.

The assay results for both hormones were expressed as ng/mL for plasma and as pg/mg for hair.

### 2.5. Statistical Analyses

Statistical analyses were performed using the software R 3.0.3 (The R Foundation for Statistical Computing). All data were tested for normal distribution by means of the Shapiro-Wilk test.

Descriptive statistics were calculated and expressed as means, standard deviations, and min/max values. Comparisons between the pre- and post-rut groups were performed by means of a non-parametric Kruskal-Wallis test. Outliers values were identified as values smaller than the lower quartile minus 3 times the interquartile range, or larger than the upper quartile plus 3 times the interquartile range. In order to evaluate the relationships between the different analyzed parameters, a non-parametric Spearman rank correlation test was performed. The statistical significance was set at *p* < 0.05 (95% C.I.). 

## 3. Results

The results of the descriptive statistics are shown in Table 1.

**Figure 1:**
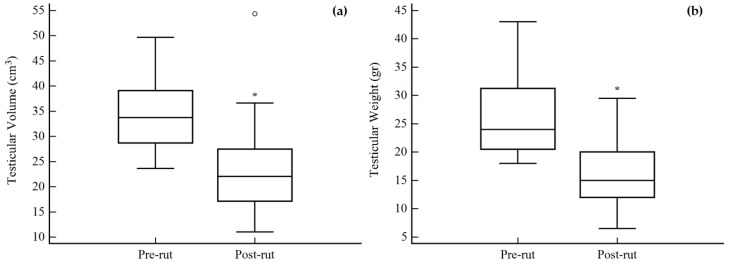
Differences between pre- and post-rut groups for the testicular morphometric parameters. (**a**) Testicular volume; (**b**) testicular weight. ○ outlier; * *p* < 0.05.

**Figure 2:**
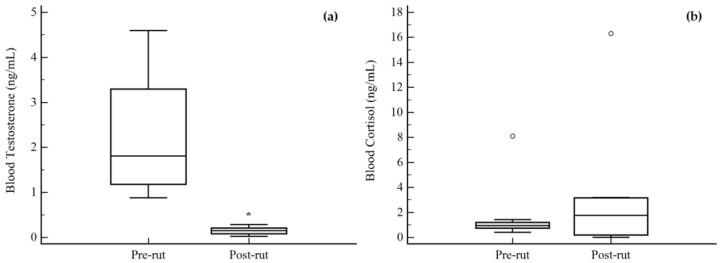
Differences between pre- and post-rut groups for the blood steroid hormone concentrations. (**a**) Testosterone; (**b**) cortisol. ○ outlier; * *p* < 0.05.

The differences between pre- and post-rut groups are reported in Figure 1 for the testicular morphometric parameters, and in Figure 2 for blood hormones levels. Testicular volume (Figure 1a) and weight (Figure 1b) significantly decreased in the post-rut group (*p* = 0.0089 and *p* = 0.0056, respectively); the same trend was noted for blood TEST concentrations (*p* = 0.0008, Figure 2a). Blood CORT levels (Figure 2b) did not show any statistical difference between the two groups (*p* = 0.8336).

**Figure 3:**
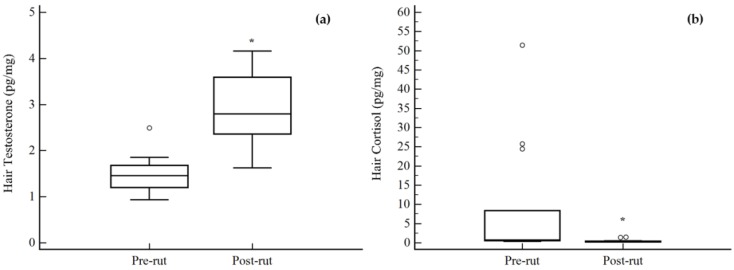
Differences between pre- and post-rut groups for the hair steroid hormone concentrations. (**a**) Testosterone; (**b**) cortisol. ○ outlier; * *p* < 0.05.

Regarding hair analyses (Figure 3), the results of the Kruska-Wallis test between the two groups showed statistical differences for both analyzed hormones. Figure 3a represents TEST concentrations, which significantly increased in the post-rut period (*p* = 0.00003), while Figure 3b shows an opposite trend, again statistically significant, for CORT (*p* = 0.0014).

Table 2 shows the correlation coefficients (ρ) between the analyzed parameters. Hair TEST concentrations were correlated to every parameter except for blood CORT (ρ = −0.07; *p* = 0.8115), while hair CORT was only correlated with hair TEST (ρ = −0.56; *p* = 0.0019). Blood TEST, testicular volume, and testicular weight correlated with each other; blood CORT was the only parameter that did not show any correlation.

## 4. Discussion

As previously stated, the male roe deer reproductive physiology has several peculiarities including a strong cycling pattern, with total functional blockage of the spermatogenesis influenced by season [1,5,6]. The involution/recrudescence alternation of the testes is directly related to the circulating blood levels of TEST [2,7], and the results of the present study regarding testicular weight, testicular volume, and blood TEST were in accordance with this, showing a significant decrease in the post-rut period (Figure 1 and Figure 2a) and a mutual correlation (Table 2). Indeed, low levels of TEST determine a decrease in meiotic and mitotic activity of the germ cells and involution of Leydig cells’ cytoplasmic volume and activity [1,5]. The same decreasing trend has already been reported for other sexual steroids in this species, including estradiol, progesterone, and androstenedione [8,24]. On the other hand, blood levels of CORT did not show any statistical difference between pre- and post-rut periods (Figure 2b). This finding may be explained by the fact that the release of cortisol has an ultradian pulsatile rhythm [25] and is influenced by a wide variety of factors, often unrelated to reproductive physiology, and is, for example, immediately increased in case of stressful events, such as hunting [26]. Moreover, blood cortisol has high intra-individual variations [27], proving to be unreliable unless related to a wider panel of analyses. Indeed, the best way to gain accurate information regarding long-term levels of CORT is to extend analyses to different matrices such as feces, urine, and hair [25]. Despite the fact that all the above-mentioned matrices still need in depth investigations [13], hair samples appear to be the most reliable, due to the lipophilic nature of the substances characterizing its sebum that permits the binding and accumulation of circulating CORT [28] and TEST [29] in the hair shafts.

The present study, to the best of the authors’ knowledge, represents the first report regarding the analysis and quantification of steroid hormones in the hair of the roe deer (*Capreolus capreolus*). The above RIA technique, already used for the same determinations in other mammalians’ species [14], proved to be reliable and capable of robust results. The rabbit anti-Testosterone antibody used in the present study was produced in our laboratories and guarantees a very good sensitivity and linearity in the used RIA system. The partial cross-reactivity with DHT, active metabolite of testosterone, has to be acknowledged, but, according to the authors, it does not compromise the aim of the study. Indeed, the outcome of the androgen analysis, TEST in this case, still shows strong statistical differences. 

The lack of existing reference values and nature of sampling does not allow for paired pre- and post-evaluations in the same animal, making the interpretation of the results quite challenging. Nonetheless, since the summer shedding of this species takes place in April [4], the hair samples analyzed in this study (collected starting from June) should represent steroid levels and subsequent accumulation during the preceding months. Unfortunately, as we are talking about wildlife, specific data regarding hormone accumulation rates and hair growth are not available. This might represent a limitation when interpreting data, but the hypothesis seems to be realistic in light of the chemical nature of the investigated molecules and specific literature regarding other animals [14]. Overall, the resulting trends and correlations of the analyzed parameters still provide interesting insights into the reproductive and behavioral physiology of the roe deer.

Hair TEST levels of the post-rut group were almost double the pre-rut levels, which is in complete contrast to the blood level trends. Testosterone reaches its blood peak during the rut period [8]; therefore, it is likely that high hair post-rut values are the direct reflection of high blood pre-rut values, since steroid hormones accumulate, with a temporal delay, in hair according to blood levels. This hypothesis is further validated by the inversely proportional pattern of the correlation between hair and blood TEST (ρ = −0.75). As expected, the same correlation pattern was noticed with the testicular parameters (Table 2).

The results of hair CORT showed a statistically significant decrease in the post-rut period (*p* = 0.0014). Hair CORT levels can be influenced by environmental temperature, with lower hormone values present during warmer months [14,30]. Therefore, higher levels in the pre-rut group may be related to the relatively lower temperatures during springtime. In addition to temperature, during springtime, bucks fight each other to establish and maintain territoriality [3] and therefore suffer from significant stress. Out of the 14 animals analyzed during pre-rut, three bucks showed extremely high values up to 51.47 pg/mg. It is impossible to give a specific explanation for this finding in light of the peculiar nature of CORT and the absence of individual behavioral information from each deer. Indeed, alongside the aforementioned high intra-individual variation of this hormone, it is not possible to rule out a chronic stress condition probably related to aggressive/territorial behavioral pattern. The Spearman’s correlation analysis performed between hair CORT and TEST showed an inversely proportional trend (ρ = −0.56; Figure 3). When discussing this finding, different factors need to be taken into consideration [31]: one being that androgens, for example, seem to inhibit the stress-induced levels of CORT [32]. This possible explanation does not hold true for the blood results, since no correlation (ρ = 0.07) between blood concentrations of CORT and TEST were noticed. The authors hypothesize that the correlation between hair TEST and CORT may be spurious, since both hormones are highly influenced by seasonality in an inversely proportional way. 

## 5. Conclusions

In conclusion, the present work represents a first report regarding the use of an RIA technique to quantify testosterone and cortisol in roe deer hair. This technique is relatively cheap and easy to perform, and its validation broadens the spectrum of available analytical tools for environmental, welfare, and reproductive studies in the roe deer. As already stated, it is pivotal to investigate alternative biological matrices especially in wildlife animals, where blood collection presents several limitations. Hair can be collected easily and is more stable than other biological samples if adequately stored [19]. Such procedures may be applied by several biometrical centers, with the ultimate goal of obtaining data from a broader population of animals. Lastly, it is important to acknowledge that cortisol and testosterone are not the only hormones that can be investigated by means of RIA on hair, helping to deepen the physiological knowledge of this peculiar species. 

The highlighted correlations seem to be in agreement with the existing literature regarding the metabolism and actions of these hormones. Nonetheless, further studies are required to confirm and better understand the cycling pattern of the reproductive physiology of this species.

## Figures and Tables

**Table 1:** Descriptive statistics of the analyzed parameters for both pre- and post-rut animals.

Parameter	Pre-Rut			Post-Rut		
	Mean (SD)	Min–Max	N	Mean (SD)	Min–Max	*n*
Testicular Weight g	26.2 (7.5)	18.0–43.0	12	16.6 (6.9)	6.5–29.5	10
Testicular Volume cm^3^	34.63 (7.14)	23.62–49.65	12	24.72 (12.04)	11.01–54.36	11
Blood TEST ng/mL	2.26 (1.42)	0.88–4.59	8	0.15 (0.09)	0.02–0.28	8
Blood CORT ng/mL	1.79 (2.57)	0.41–8.10	8	3.32 (5.44)	0.01–16.32	8
Hair TEST pg/mg	1.48 (0.39)	0.93–2.49	14	2.89 (0.77)	1.62–4.16	14
Hair CORT pg/mg	8.72 (15.07)	0.36–51.47	14	0.49 (0.42)	0.21–1.51	14

TEST: testosterone; CORT: cortisol.

**Table 2:** Spearman rank correlation coefficients (ρ) table with *p* values (95% C.I.) for all the analyzed parameters.

	Hair TEST	Hair CORT	Blood TEST	Blood CORT	Testicular Volume	Testicular Weight
Hair TEST		−0.56 *p* = 0.0019	−0.75 *p* = 0.0007	−0.07 *p* = 0.8115	−0.58 *p* = 0.0040	−0.57 *p* = 0.0072
Hair CORT			0.40 *p* = 0.1287	0.33 *p* = 0.2168	0.14 *p* = 0.5264	0.26 *p* = 0.2500
Blood TEST				0.07 *p* = 0.8075	0.72 *p* = 0.0037	0.76 *p* = 0.0044
Blood CORT					−0.18 *p* = 0.5468	0.00 *p* = 0.9914
Testicular Volume						0.96 *p* < 0.001
Testicular Weight						

TEST: testosterone; CORT: cortisol.
